# Non-contact and label-free biomechanical imaging: Stimulated Brillouin microscopy and beyond

**DOI:** 10.3389/fphy.2023.1175653

**Published:** 2023-03-31

**Authors:** Chenjun Shi, Hongyuan Zhang, Jitao Zhang

**Affiliations:** 1 Department of Biomedical Engineering, Wayne State University, Detroit, MI, United States,; 2 Cole Eye Institute, Cleveland Clinic, Cleveland, OH, United States

**Keywords:** biomechanics, mechanical imaging, Brillouin microscopy, stimulated Brillouin scattering, optical elastography, picosecond ultrasonics, phonon microscopy

## Abstract

Brillouin microscopy based on spontaneous Brillouin scattering has emerged as a unique elastography technique because of its merit of non-contact, label-free, and high-resolution mechanical imaging of biological cell and tissue. Recently, several new optical modalities based on stimulated Brillouin scattering have been developed for biomechanical research. As the scattering efficiency of the stimulated process is much higher than its counterpart in the spontaneous process, stimulated Brillouin-based methods have the potential to significantly improve the speed and spectral resolution of existing Brillouin microscopy. Here, we review the ongoing technological advancements of three methods, including continuous wave stimulated Brillouin microscopy, impulsive stimulated Brillouin microscopy, and laser-induced picosecond ultrasonics. We describe the physical principle, the representative instrumentation, and biological application of each method. We further discuss the current limitations as well as the challenges for translating these methods into a visible biomedical instrument for biophysics and mechanobiology

## 1 Introduction

Biomechanical properties of cell and tissue are playing crucial roles in many aspects of physiological and pathological conditions [1–11]. Therefore, quantification of biomechanical properties becomes an essential need in the field of biophysics and mechanobiology [12, 13]. Among all existing techniques, non-contact optical elastography is favorable because they can conduct 2D/3D mechanical measurement without physically disturbing the native condition of the sample [14, 15]. Confocal Brillouin microscopy [16, 17] (CBM) is an emerging optical elastography technology that solely uses a laser beam to probe the high-frequency 3D elastic longitudinal modulus with a diffraction limited resolution (<1μm). In the past decades, technology innovation enables quickly expanding applications of the CBM in many biomedical fields, including cellular biomechanics, developmental biology, ocular diseases, tumorigenesis, angiogenesis, biomaterial characterization, and other medical diagnoses [18]. The state-of-the-art instruments of CBM and the applications have been well documented in recent review articles [19–22]. Currently, the acquisition speed of CBM is around 50–100 ms per pixel [18], which is much slower than other imaging modality such as fluorescence confocal microscope and is one of major obstacles for real-time mechanical imaging. The fundamental reason for the slow speed is that the CBM is based on the spontaneous Brillouin scattering [23], which is a linear optical process and has extremely low scattering efficiency (i.e., the ratio of the powers of the scattered light and the incident light is about 10^−9^). As a result, the Brillouin spectrometer requires long exposure time to collect enough scattered photons for signal analysis. Another limitation of the existing CBM is the spectral broadening, which is mainly introduced by the frequency-domain optical spectrometer (a recently proposed heterodyne detection scheme could potentially avoid the spectrum broadening of the spectrometer [24].). Due to the limited spectral resolution of the dispersion devices (i.e., FabryPerot etalon, or virtually imaged phased array) used in the spectrometer, the intrinsic instrumental linewidth is ~100 MHz to ~600 MHz [25,26]. As such, the linewidth of the detected signal will be broadened because of the convolution of the signal and the instrumental line shape. In case the sample within the optical voxel is homogeneous, the natural linewidth can be extracted by deconvolution process with the known instrumental line shape [27], but the possible error introduced by the measurement of instrumental line shape needs to be carefully characterized. Since the linewidth of the Brillouin signal is associated with the acoustic attenuation thus the viscous properties of the material [28, 29], the broadening effect can greatly reduce the sensitivity of Brillouin technology for quantifying the viscosity of material, leaving this field mostly undeveloped.

Several approaches have been developed to improve the performance of Brillouin technology, which can be loosely classified into three types: i) Spontaneous scattering-based method. Since spontaneous Brillouin scattering runs in a nondepleted pump condition, reuse of non-scattered pump light for extra pixels can significantly reduce the overall acquisition time in 2D/3D mapping. To this end, line-scanning Brillouin microscopy (LSBM) has been developed by acquiring Brillouin signals of multiple pixels along a line with a multiplexed spectrometer [30]. Although the acquisition time for each pixel is similar to that of a standard CBM, the multiplexing design in the LSBM allows more than 100 pixels to be measured in one shot. Consequently, the acquisition time for 2D/3D biomechanical imaging was reduced by more than one order of magnitude [31,32]. ii) Stimulated scattering-based methods. Different from spontaneous Brillouin scattering where the acoustic phonons are from thermally induced density fluctuation of the material, the stimulated scattering-based methods utilize optically-excited coherent phonons for Brillouin scattering and can significantly enhance the Brillouin signal [23], which alternatively can shorten the acquisition time if maintaining the signal to noise ratio (SNR). These include continuous wave stimulated Brillouin scattering (cW SBS) [23], impulsive stimulated Brillouin scattering [33,34], and laser induced picosecond ultrasonics [35]. iii) Brillouin enhancement techniques. These techniques have been developed to further improve the SNR for either spontaneous or stimulated scattering process, including ultrasonic enhancement [36] and quantum enhancement [37].

Traditionally, stimulated Brillouin scattering specifically referred to the non-linear photon-phonon interaction caused by electrostriction and/or optical absorption when an intense optical field is presented [23, 38]. Here, we broadly consider a Brillouin scattering as a stimulated process if the phonons are generated by the presence of a light field, in contrast to the spontaneous process where phonons are from the inherent thermal fluctuations. Comparing with spontaneous scattering, stimulated Brillouin scattering process has much higher scattering efficiency; thus, it is promising to push Brillouin technology to a new level, where the acquisition speed of a Brillouin microscope is comparable with that of a fluorescence confocal microscope. In addition, as the frequency-domain optical spectrometer is not required for signal detection, stimulated Brillouin scattering-based methods could achieve a much better spectral resolution for linewidth measurement, paving the way for non-contact quantification of both elasticity and viscosity of material [39]. In recent years, ideas and techniques of stimulated scattering-based methods are being adapted for biomedical research and demonstrating encouraging feasibility in quantifying cell and tissue biomechanics. In this Review, we first introduce the physical basis of several stimulated Brillouin techniques. We then discuss the latest progress on instrumentation and biomedical applications. In the end, we discuss existing challenges in translating stimulated Brillouin technology into a visible mechanical imaging modality for biomedical research.

## 2. Principle and instrumentation

### 2.1. Continuous wave stimulated Brillouin microscopy (cw SBM)

**FIGURE 1:**
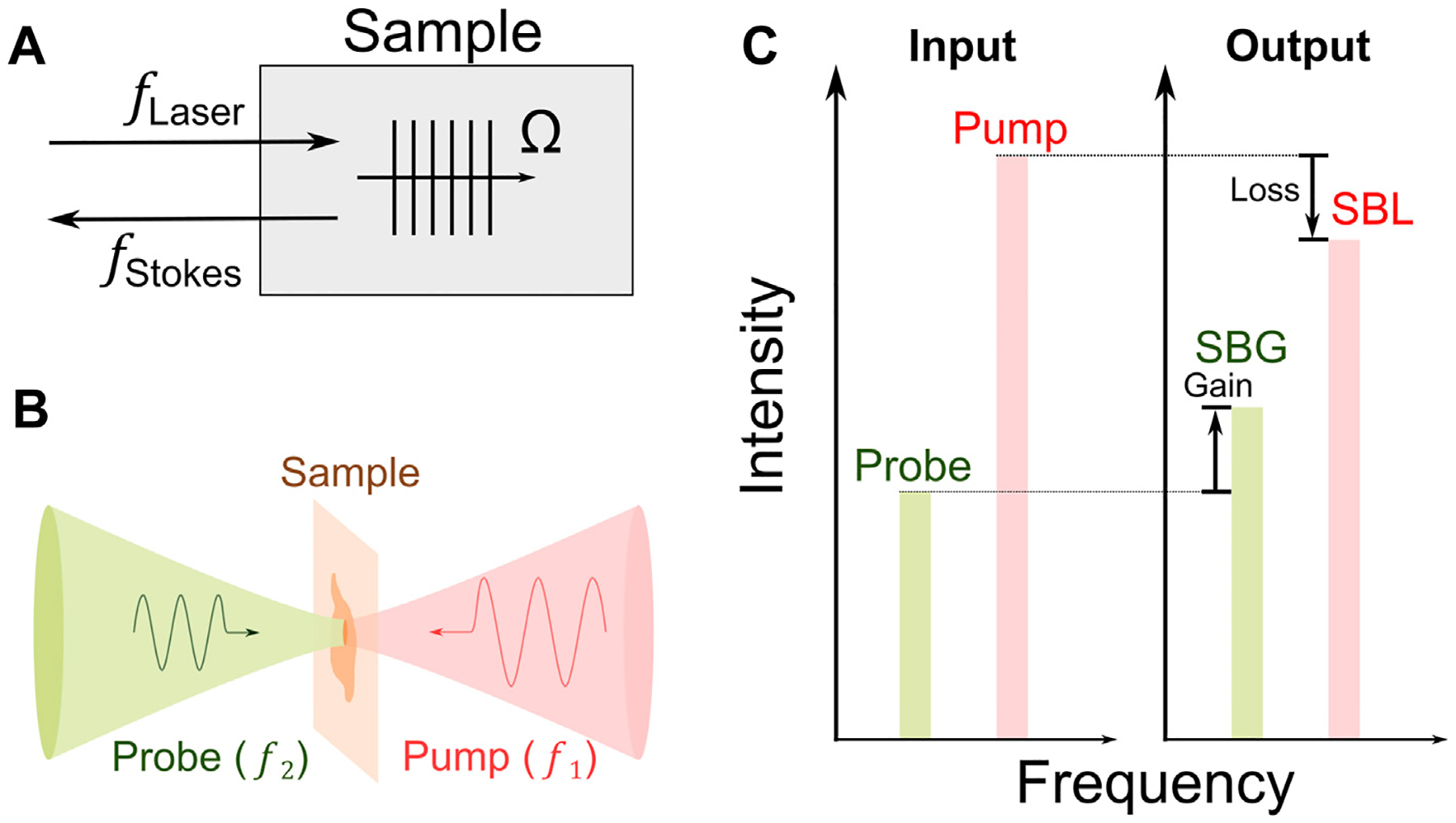
Principle of stimulated Brillouin scattering. (A) Schematic of stimulated Brillouin scattering process. Ω: frequency of acoustic wave. (B) Scheme of cw SBS used for biomedicine. (C) Energy transfer between the pump and the probe. SBG: stimulated Brillouin gain; SBL: stimulated Brillouin loss.

In 1964, stimulated Brillouin scattering (SBS) was first observed by focusing a high-energy pulse laser into an optical crystal [38]. In SBS, the intense incident light field generates a backward propagating Stokes light after scattered by the acoustic wave within the material (Figure 1A). Ultimately, the coherent interaction among the incident light, the Stokes light, and the acoustic wave will significantly enhance the scattered Stokes light in a non-linear manner [23]. For biomedical application, the SBS is currently established by two cw laser beams to mitigate the severe photodamage caused by the intense laser field (Figures 1B,C). A high-power pump beam $\left(f_{1}\right)$ and a low-power probe beam $\left(f_{2}\right)$ are counterpropagating in the sample. Once the frequency difference $\Delta f=f_{1}-f_{2}$ matches the frequency of the acoustic phonon (i.e., Brillouin shift of the material) $f_{B}\left(=\Omega \right)$, the intensity of the transmitted probe beam is amplified by the SBS process:

(1) $$I_{2}\left(\Delta f\right)=I_{2}\cdot exp\left(g\left(\Delta f\right)I_{1}L\right)$$

where $I_{1}$ and $I_{2}$ is the incident power of pump and probe beam, respectively. L is the interaction length of two beams within the sample (for microscopic setup, it is usually determined by the size of the focused beam voxel), and g is the gain factor. As a result, the relative gain (i.e., SBG) of the probe beam is

(2) $$G\left(\Delta f\right)=\frac{I_{2}\left(\Delta f\right)-I_{2}}{I_{2}}=exp\left(g\left(\Delta f\right)I_{1}L\right)-1$$

and

(3) $$g\left(\Delta f\right)=g_{0}\cdot \frac{{\left(\Gamma_{B}/2\right)}^{2}}{{\left(f_{B}-\Delta f\right)}^{2}+{\left(\Gamma_{B}/2\right)}^{2}}$$

with $\Gamma_{B}$ : Brillouin linewidth (full width of half maximum), and $g_{0}$ : line-center gain factor.

(4) $$g_{0}=\frac{4\pi^{2}\gamma_{e}^{2}f^{2}}{nvc^{3}\rho \Gamma_{B}}$$

where $\gamma_{e}$ : electrostrictive constant; $f\left(\approx f_{1},f_{2}\right)$: frequency of light; n and ρ : refractive index and density of the material; v: speed of sound, c: speed of light; Eqs. 2–3 indicate that the relative gain of the probe beam G(Δf) has the peak value once $\Delta f=f_{B}$.

In experiment, with the measured spectrum of G(Δf), the Brillouin shift $f_{B}$ and the line-center gain factor $g_{0}$ can be determined. According to the principle of Brillouin scattering, Brillouin shift $f_{B}$ at 180° scattering geometry is related to the complex longitudinal modulus $M=M^{\prime }+i\cdot M^{\prime \prime }$ by

(5) $$f_{B}=\frac{2nf}{c}\cdot v=\frac{2nf}{c}\cdot \sqrt{\frac{M^{\prime }}{\rho }},$$

(6) $$M^{\prime \prime }=\frac{c^{2}}{4n^{2}}\cdot \frac{\rho \cdot f_{B}\cdot \Gamma_{B}}{f^{2}}$$

where $M^{\prime }$ and $M^{\prime \prime }$ are the storage modulus and loss modulus, respectively. Thus, the density of the material can be determined based on Eq. 4

(7) $$\rho =\frac{2\pi^{2}\gamma_{e}^{2}f^{3}}{c^{4}f_{B}g_{0}\Gamma_{B}}$$

In short, the cw SBS process allows us to directly probe the Brillouin shift, the longitudinal modulus, and the density of the material.

**FIGURE 2:**
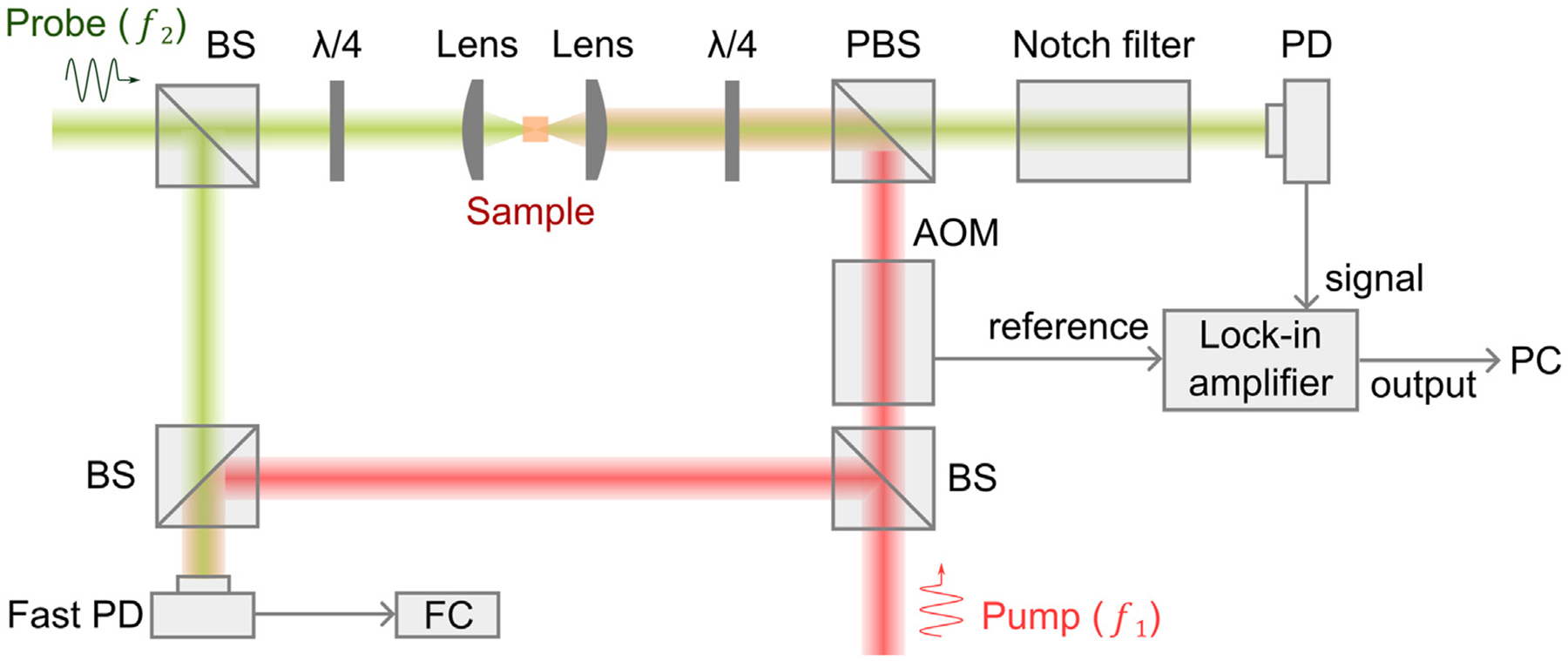
Schematic of a representative cw SBM setup. λ/4: quarter-wave plate; PBS: polarized beam splitter; BS: beam splitter; AOM: acousto-opticmodulator; PD: photodetector; FC: frequency counter; PC: computer

A representative optical configuration of the cw SBM is shown in Figure 2 [39–41]. Two tunable lasers with narrow linewidths (~MHz or smaller) and 10–50 GHz tunable ranges are used for generating pump and probe beams. The frequency of the pump beam is locked to a frequency standard (e.g., Rb85 absorption line), and the frequency of the probe laser is scanned by controlling electronics. The two beams counter propagate with a geometry of ~180^∘^ and overlap at the common focal plane of two identical objective lenses. The readout of the transmitted probe beam is achieved by manipulating its polarization. For high-sensitivity detection, the pump beam is modulated by an acousto-optic modulator at ~1 MHz, and a lock-in amplifier is used to extract the relative gain of the probe beam. To suppress reflected stray light of the pump beam, a notch filter (e.g., Rb85 gas cell) is placed in front of the photodetector. To monitor the frequency difference of the pump beam and the probe beam, a small portion of two beams is picked for frequency beating, and the beat frequency is measured by a frequency counter. In addition, a white light beam can be coupled into the optical path for colocalized brightfield imaging.

For sample testing, the pump beam and the probe beam run at a power of 100–~300 mW, and 10–45 mW, respectively. Under this power level, an acquisition time as short as 2 ms has been achieved for water sample, and 20 ms for biological sample [39]. This is comparable to the state-of-the-art spontaneous CBM [18, 42]. The reason that the current cW SBM does not show significant improvement against CBM is probably due to the non-optimized operation of the setup. It has been demonstrated that the relative gain of the probe beam is about 10^−6^, which is higher than spontaneous scattering but still in the linear region of the stimulated scattering [43]. In addition, the suboptimized technical parameters for signal detection limit the performance of cW SBM. Technical advances are being made to address this issue. For example, a very recent work shows ten times improvement in the acquisition speed can be achieved by optimizing the detection using localization theory [40]. Furthermore, the efficiency of SBM can be improved by replacing cw operation with quasi-pulsed operation [44]. By optimizing the pump-probe interaction at high peak power and short interaction time, the quasi-pulsed SBM can reduce the illumination power by 20 times while maintaining the signal quality. As the signal of Cw SBM is obtained by scanning the frequency of the laser, the spectral resolution is ultimately limited by the intrinsic linewidth of the laser source as well as the frequency dispersion during scanning. In experiments [39], cW SBM can achieve a spectral resolution of 39 MHz with mediate numerical aperture (NA), which is 2–20 times better than the Brillouin spectrometers of CBM. This enables cw SBM to detect mechanical features with much better specificity.

To date, the feasibility of cw and quasi-pulsed SBMs has been demonstrated in liquid (e.g., water, methanol, intralipid solution) [43, 45], C. elegans [39], single cell, and organoids with microscopic resolution [44], suggesting promising potential of SBM as a new biomechanical imaging modality. Importantly, in addition to the Brillouin shift and linewidth, cw SBM can probe the density of the material. This will not only provide additional contrast mechanism for biophysical imaging but also allow direct quantification of longitudinal modulus of some biological cells and tissue in which the refractive index and density are dependent on each other [46–48].

### 2.2. Impulsive stimulated Brillouin microscopy (ISBM)

**FIGURE 3:**
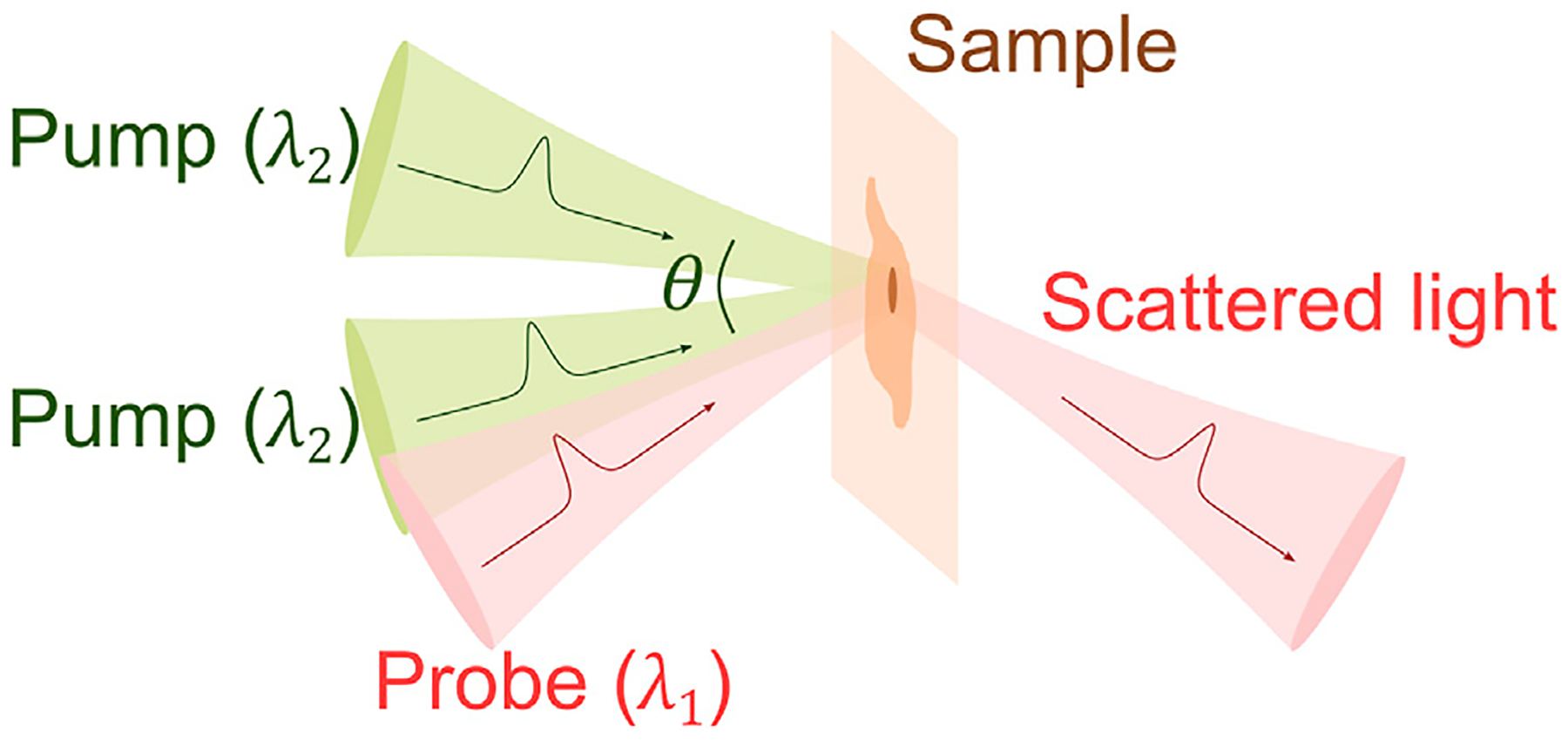
Principle of ISBS

The impulsive stimulated Brillouin scattering (ISBS) is also known as “laser induced phonons” or “laser induced dynamic grating” [34, 49–52], which was initially developed for studying phonon-phonon interaction in molecular crystals around 1980s. A representative scheme of ISBS is shown in Figure 3. Two timecoincident pulsed laser beams (i.e., pump beams) are crossed in the sample at an angle of θ. The optical interference pattern generated at the overlapping region creates a transient density grating due to electrostrictive excitation and/or thermal excitation [34, 53]. This excited density grating can be interpreted as counterpropagating acoustic waves whose wavelength (Λ) follows the period of the grating. For an optical isotropic material, it has the form of

(8) $$\Lambda =\frac{\lambda }{2sin\left(\theta /2\right)}$$

where λ is the wavelength of the pump beam, and θ is the angle between the two crossed beams. The excited acoustic wave is then probed by a third beam (i.e., probe beam) at the phase-matching angle for Bragg diffraction. The properties of the excited acoustic wave can then be extracted by detecting the diffracted light of the probe beam with a photodetector. In case the transient density grating is caused by electrostriction, at Bragg condition, the diffraction efficiency η(t) of the probe beam can be expressed as [54]

(9) $$\eta \left(t\right)\approx {\left(\frac{\pi n^{6}k_{s}p^{2}I_{\text{pump}}\cdot L}{\lambda_{\text{probe}\cdot \;}v\rho c}\right)}^{2}exp\left(-2\gamma_{s}t\right)sin^{2}\left(\omega_{s}t\right)$$

where n is refractive index of the sample, $k_{s}$ is the wavevector of the acoustic phonon, p is the optoelestic constant that is dependent on the polarization of pump beam, $I_{\text{pump}}$ is the energy of each pump pulse, L is the effective grating thickness, $\lambda_{\text{probe}}$ is the wavelength of the probe beam, v is the speed of sound, ρ is the density of the sample, c is the speed of light in air, $\gamma_{s}$ is the acoustic attenuation constant, $\omega_{s}$ is the angular frequency of the acoustic phonon. Eq. 9 indicates that the diffracted light of the probe beam is proportional to the square of the pulse energy of the pump beam, which has been observed in experiment [55]. Although the pump beam needs to be ultrashort pulses, the probe beam can be either a cw laser or a pulsed laser [50,56]. The choice depends on the time scale of the dynamic process within the material: for slow process that is within the bandwidth of detection electronics, a cw laser can be used. Otherwise, a pulsed laser and a delay line are necessary to extract the signal. To improve the detection sensitivity, an optical heterodyne scheme was developed [57, 58], where an optical transmission grating was introduced to produce two pump beams, a probe beam, and a reference beam using the first (±1) diffraction orders. This optical arrangement significantly simplified the optical alignment for heterodyne detection. Therefore, it has become a standard design for ISBS and is widely adopted nowadays. Under the scheme of heterodyne detection, the detected signal is linearly related to the pulse energy of the pump beam [59].

**FIGURE 4:**
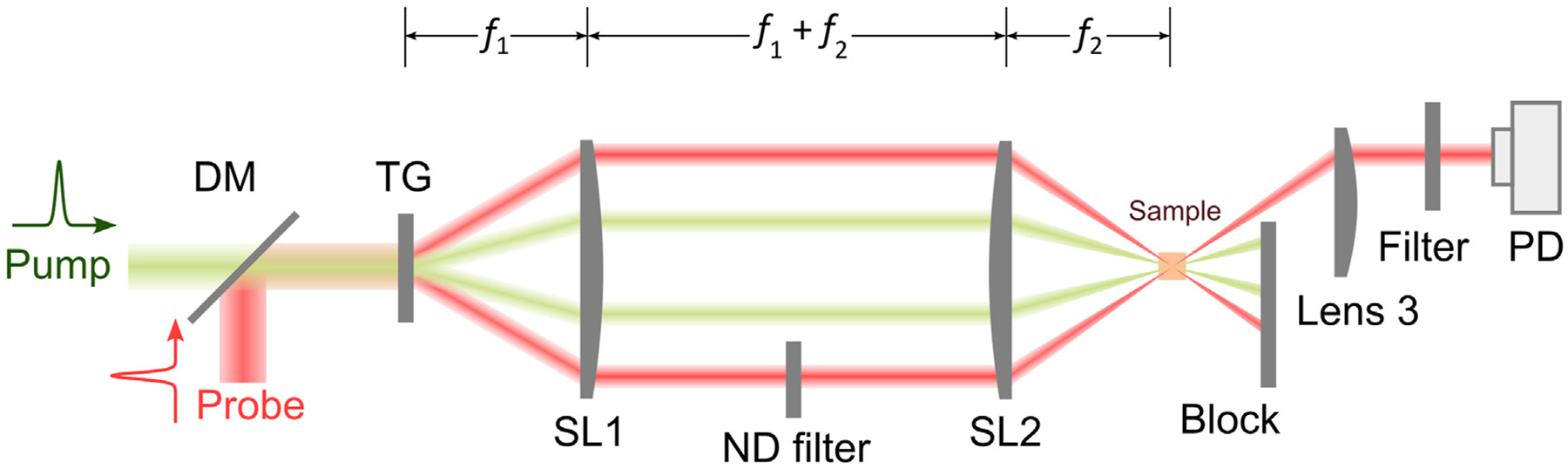
Schematic of a representative ISBM setup. DM: dichroic mirror; TG: transmission grating; PD: photodetector. $f_{1},f_{2}$: focal length of Lenses SL1 and SL2, respectively.

In recent years, there are increasing interests in adapting ISBS for biomedical applications [55, 60–62]. A representative schematic of the ISBM setup is shown in Figure 4. The pump laser is around 500 nm and has a pulse width of ~1–10 ps. The probe laser is a cw laser and has a longer wavelength (e.g., 780 nm or 895 nm). The two laser beams are coupled into an optical transmission grating using a dichroic mirror. The four beams after the optical grating are delivered to the sample with a 4f telescope system. The diffracted light of the probe beam is then detected by a photodetector. After filtering and amplification, the signal is collected by an oscilloscope, which is triggered by the signal from the pump laser. For heterodyne detection, the intensity of the reference beam is attenuated to a proper level by a neutral density filter. The frequency of the acoustic wave (i.e., Brillouin shift) can be extracted by conducting Fourier analysis of the collected time-domain signal. For very weak signal, a new technique for spectral analysis named “adaptive noise-suppression Matrix Pencil” was recently reported to improve the detection sensitivity [63]. In addition, the wavelength (Λ) of the acoustic phonon can be derived from the period $\left(d_{T}\right)$ of the optical grating based on the 4f system:

(10) $$\Lambda =d_{T}\cdot f_{2}/f_{1}$$

where $f_{1},f_{2}$ is the focal length of the lens SL1 and SL2, respectively. This further allows us to quantify the speed of sound in the material. Due to the large wavelength of the excited phonons (e.g., ~10μm), the measured Brillouin shift of current ISBM is around ~100 MHz, one order of magnitude lower than that of CBM and cw SBM. This also sets a limit for the spatial resolution of ISBS-based imaging [62, 64]. The first microscopic imaging of liquid samples using ISBM was reported in 2017 [61]. Later, ISBS experiments were conducted on biologically relevant hydrogels with acquisition time of sub-ms [55], indicating an encouraging future of ISBM for biomedical application.

### 2.3. Laser-induced picosecond ultrasonics

**FIGURE 5:**
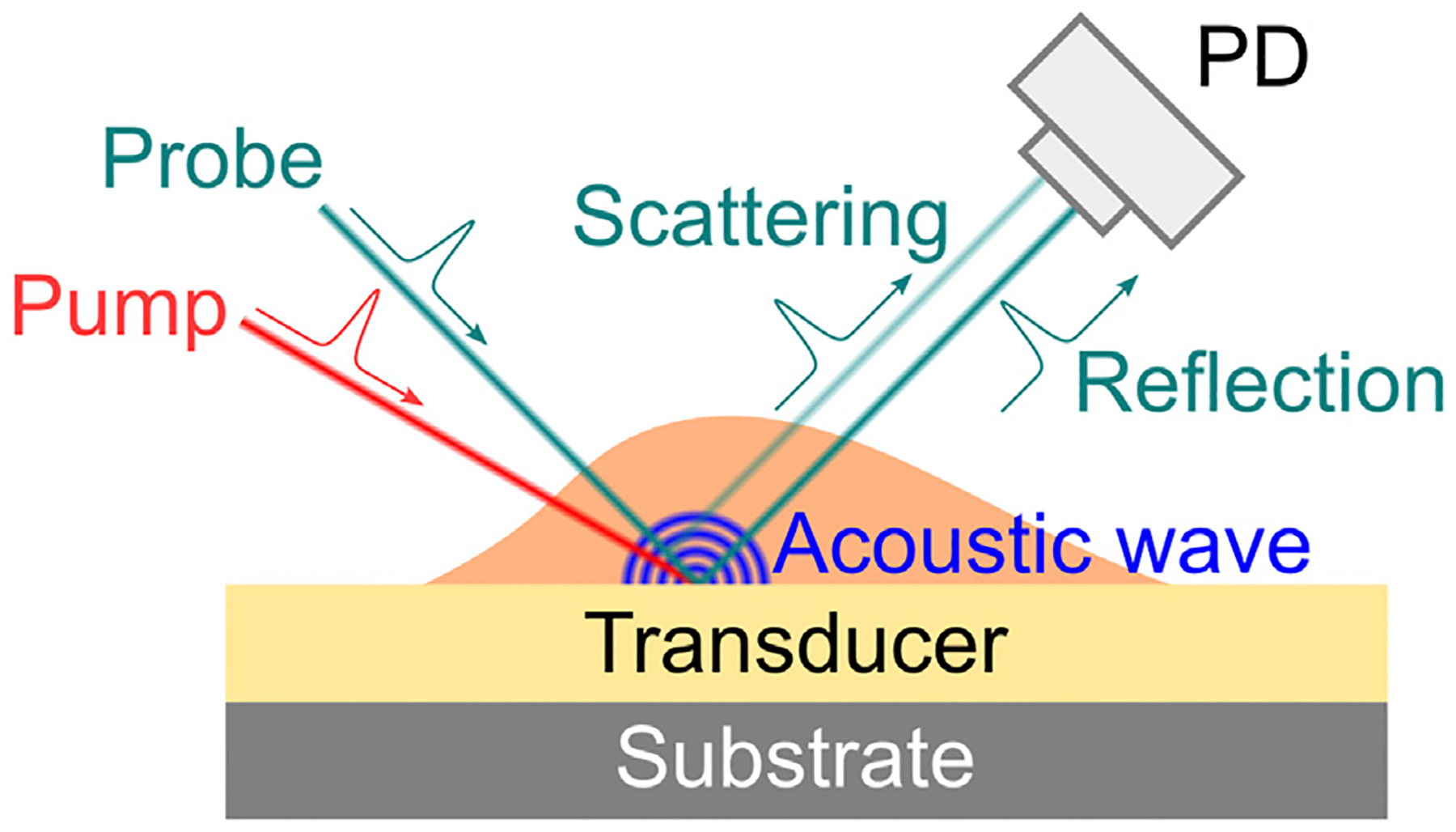
Principle of laser-induced picosecond ultrasonics. PD: photodetector. The angles of beams are for the purpose of demonstration only. Normal illumination/detection is usually used in experiment.

Picosecond ultrasonics (PU) is another way to excite coherent phonons for Brillouin scattering. Different from cw SBM or ISBM where the phonons are directly generated within the sample by a laser beam, PU excites coherent phonons with the help of an ultrasonic transducer (usually an absorbing film on a substrate) (Figure 5). When a femtosecond laser pulse (i.e., pump beam) shines on the transducer, a picosecond acoustic wave will be launched because of the thermoelastic expansion induced by the absorption of the laser pulse [65,66]. As a sample is present on top of the acoustic transducer, the propagation behavior of the acoustic wave within the sample can be accessed by a second laser pulse (i.e., probe beam) based on the pump-probe technique. With normal incidence, the backward scattered Brillouin signal is detected by the interference of the scattered light and the reflected probe light at the photodetector. The frequency information can then be extracted from the temporal signal using frequency analysis [67,68]. Since the detected signal is a time-domain trace, the PU is also called the time-domain Brillouin scattering [35].

One distinct feature of the PU is that its axial resolution can be dominated by the acoustic wavelength instead of the optical depth of focus. According to the Brillouin scattering at 180^∘^ geometry $\left(f_{B}=2nv/\lambda_{\text{probe}}\right)$, the acoustic wavelength $\lambda_{a}$ is determined by

(11) $$\lambda_{a}=\lambda_{\text{probe}}/2n$$

where $\lambda_{\text{probe}}$ is the wavelength of the probe beam, and n is the refractive index of the material. As $\lambda_{a}$ is shorter than the optical wavelength, the axial resolution of the PU could be better than the optical depth of focus. The Brillouin frequency $f_{B}$ of the PU is time dependent. As a result, its spatial location can be determined by the speed of sound v (calculated from the measured Brillouin frequency and the known refractive index). Therefore, the axial sectioning can be achieved by analyzing the instantaneous frequency of the time-domain signal without physically scanning the beam spot along axial direction [69]. The axial resolution of the PU is estimated to be

(12) $$\Delta z=N\cdot \lambda_{\text{probe}}/4n$$

where the integer N is the number of complete cycles of acoustic wave used for frequency analysis. In practice [67,69],N=4 or 6 were used for biological imaging, yielding an axial resolution of less than 1μm with a low NA (i.e., 0.4–0.6) objective lens. Meanwhile, the lateral resolution of the PU is still optical diffraction limited.

**FIGURE 6:**
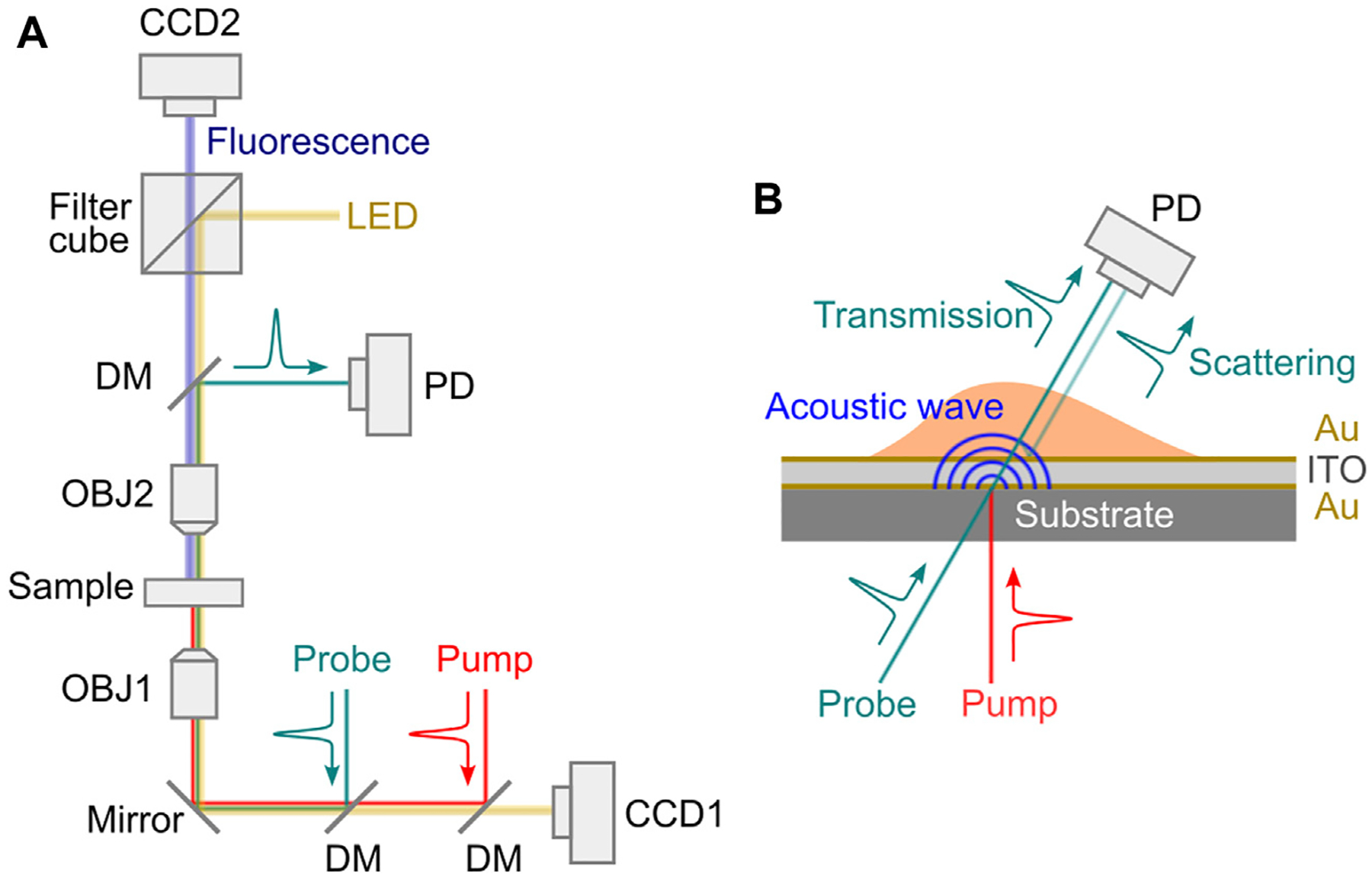
Schematic of laser-induced picosecond ultrasonics setup. (A) Representative optical setup. LED: light emitting diode; DM: dichroic mirror; OBJ1-OBJ2: objective lens; PD: photodetector. (B) Transducer design.

**FIGURE 7:**
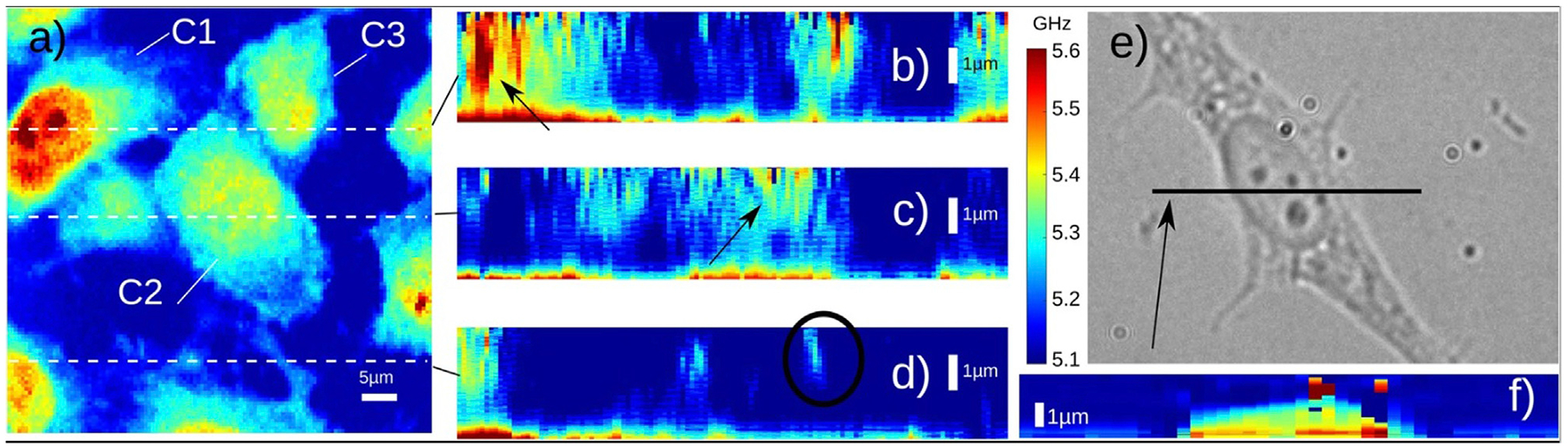
Brillouin imaging of fixed 3T3 cells. (A) Three cells (C1–C3) are shown in the image. (B–D) Depth images of three locations (indicated by the dashed lines in (A) Black circle indicates the location of filopodia. (E–F) Bright-field image and Brillouin image of a 3T3 cell. Adapted from Ref. [69].

The PU technology was developed in 1980s for studying the propagation behavior of high-frequency phonons in material [65]. In recent years, there are increasing demonstrations of this technology with both fixed and live biological cells [67–75]. Representative instrument of the PU technology (also called phonon microscopy) [67] is shown in Figure 6A. The pump and the probe lasers have the wavelength of 390 nm and 780 nm, respectively. Both lasers have a pulse width of 150 fs and a repetition rate of ~100 MHz. An asynchronous optical sampling technique was used for setting the time delay between the two lasers, so that a mechanical delay line is not required. The setup works in a transmission mode: the pump and the probe beam were focused into the sample with objective lenses, and the transmitted probe beam as well as the scattered light were detected by a photodetector. A LED light source was coupled into the setup for colocalized brightfield and fluorescence imaging. The design of the acoustic transducer was shown in Figure 6B. It has a sandwiched layer structure deposited on a sapphire substrate: Au/ITO/Au. The thickness of each layer was determined by considering: 1) the probe beam can efficiently pass through while the pump beam is mostly absorbed by the bottom Au layer; and 2) the acoustically resonant frequency of the transducer can be easily separated from the Brillouin frequency of the sample [74]. The backward scattered light of the transmitted probe beam will be reflected by the top Au layer and interfere with the probe beam itself at the photodetector. To conduct biological experiments (Figure 7), cells were placed on top of the transducer, and the average power of 0.4 mW and 1 mW was used for the pump and the probe beam, respectively. Considering the transmission of the transducer, about 0.3 mW was ultimately illuminated into the cell body. The acquisition time for each pixel is about 1–2 s. With the collection objective lens of 0.42 NA, the PU microscope can achieve axial resolution of ~0.8 um for cellular samples [67,69], which is a few times smaller than the optical depth of focus. In addition to the optical scheme in free space, the PU technology was recently demonstrated in a fiber-based setup [76], suggesting its potential as an endoscopy.

One potential limitation of the PU technology is the temperature rise at the transducer-cell interface. Both simulation [67] and experiment [68] suggest an increase of 7°C-13°C above room temperature during the steady-state running. This may not only affect the biological function of live cells but also introduce artifacts to the measured Brillouin shift because it is temperature dependent. Another limitation is the depth of the imaging. Because high-frequency acoustic wave rapidly attenuates as it propagates, the detection of the PU signal should take place in the vicinity of the transducer, typically within 10 μm [74].

## 3. Summary and outlook

Brillouin scattering based elastography is an attractive toolbox for biophysics and mechanobiology because it provides a non-contact and label-free approach to probing biomechanics with (sub)cellular resolution in 3D. Although the confocal Brillouin microscopy is being recognized as an emerging tool in several aspects of biomedical research, one obstacle that hinders the wide adoption of this technology is the slow acquisition speed. To this end, stimulated Brillouin based techniques provide a promising future because they can generate Brillouin signal more efficiently by utilizing optically excited coherent phonons. In addition, the natural linewidth and the density of the material can be probed by Cw SBM, which not only provides additional contrast mechanisms for mechanical imaging but also allows the direct quantification of longitudinal modulus under some circumstances.

In this Review, we introduced three variants of stimulated Brillouin based technology and summarized their technical features as well as performances. As shown in Table 1, the recent technological advance is certainly encouraging. Meanwhile, there are still limitations and challenges. First, the benefit of efficient Brillouin scattering in stimulated process is not yet reflected in the acquisition speed of the instrument. Currently, the acquisition speed of stimulated Brillouin is comparable to spontaneous Brillouin. This is mainly due to the less optimized signal detection. For cw SBM, an additional reason is that the current setup works in the weak non-linear regime thus has limited gain. Second, most of the existing setups work in the transmission mode, limiting themselves to thin, transparent, and/or homogeneous samples. However, many biological samples such as cell cluster and tissue are thick, translucent, and heterogeneous. To this end, an instrument working in reflection mode would be necessary. Third, the potential phototoxicity and the temperature-related artifacts caused by the high-power laser are not well characterized. In Cw SBM, the continuous operation of the lasers not only limits the performance of the instrument but also cause considerable phototoxic stress to the sample. Ideally, a pulsed operation of SBM could significantly mitigate the above issue but requires comprehensive consideration of the laser parameters and the availability of the laser source. In PU, the significant temperature rise at the transducer-sample interface may alter the physiological condition of the sample and introduce artifact to the result. To avoid the temperature rise, thermal insulation method could be considered in the design of the acoustic transducer. In the future, with substantial technical innovations that overcome existing challenges, the stimulated Brillouin based technology is promising to become a visible instrument for biomechanical research.

## Figures and Tables

**TABLE 1:** Comparison of the representative stimulated Brillouin based instruments.

Technique	Laser source	Laser power (mw)	Wavelength (nm)	Acquisition time/pixel	Best resolution (μm)	SNR	Biological application
cw SBM [39, 40, 43]	cw	100–300 (pump)	780	2–20 ms	0.3 × 2	~70^a^	*C. elegans*
		10–45 (probe)					
ISBM [22, 55, 61]	pulsed + cw	10–400 (pump)	~500 (pump)	0.3 ms	10 × 230	8^b^	hydrogel
		20–41 (probe)	780/895 (probe)				
PU [67, 68]	pulsed	0.4–7.6 (pump)	390/415 (pump)	1–2 s	1.1 × 0.8	40–70^c^	fixed and live cells
		1–2 (probe)	780/830 (probe)				

a Note1 [40]: SNR was measured by the ratio between the mean and standard deviation of SBG peak intensity after curve fitting. Sample: water; pump power: 250 mW; probe power: ~20 mW; acquisition time: 20 ms

b Note2 [61]: Not specify how SNR was quantified. Sample: saturated brine solution; pump power: 400 mW (pulse energy: ~40 μJ, repetition rate: 10 kHz); probe beam: 41 mW; acquisition time: 0.2 ms.

c Note3 [67]: SNR was measured by the ratio of the peak amplitude of the acoustic signal to the standard deviation of the noise background in the absence of signal in the same band. Substrate was not specified. Pump power: 0.5 mW; probe power: 1 mW; acquisition time: 0.2 ms.

## References

[1] Vogel V, Sheetz M. Local force and geometry sensing regulate cell functions. Nat Rev Mol Cel Biol (2006) 7(4):265–75. doi:10.1038/nrm1890 16607289

[2] Wang N, Tytell JD, Ingber DE. Mechanotransduction at a distance: Mechanically coupling the extracellular matrix with the nucleus. Nat Rev Mol Cel Biol (2009) 10(1): 75–82. doi: 10.1038/nrm2594 19197334

[3] Davidson L, von Dassow M, Zhou J. Multi-scale mechanics from molecules to morphogenesis. Int J Biochem Cel Biol (2009) 41(11):2147–62. doi:10.1016/j.biocel.2009.04.015 PMC2753763 19394436

[4] Wirtz D, Konstantopoulos K, Searson PC. The physics of cancer: The role of physical interactions and mechanical forces in metastasis. Nat Rev Cancer (2011) 11(7): 512–22. doi:10.1038/nrc3080 21701513 PMC3262453

[5] Miller CJ, Davidson LA. The interplay between cell signalling and mechanics in developmental processes. Nat Rev Genet (2013) 14(10):733–44. doi:10.1038/nrg3513 24045690 PMC4056017

[6] Gilmour D, Rembold M, Leptin M. From morphogen to morphogenesis and back. Nature (2017) 541(7637):311–20. doi:10.1038/nature21348 28102269

[7] Nikolopoulou E, Galea GL, Rolo A, Greene ND, Copp AJ. Neural tube closure: Cellular, molecular and biomechanical mechanisms. Development (2017) 144(4): 552–66. doi:10.1242/dev.145904 28196803 PMC5325323

[8] Chaudhuri PK, Low BC, Lim CT. Mechanobiology of tumor growth. Chem Rev (2018) 118(14):6499–515. doi:10.1021/acs.chemrev.8b00042 29927236

[9] Moon LD, Xiong F. In Mechanics of neural tube morphogenesis. Seminars in cell and developmental biology. Elsevier (2021). 10.1016/j.semcdb.2021.09.009 34561169

[10] Nguyen LT, Jacob MAC, Parajón E, Robinson DN. Cancer as a biophysical disease: Targeting the mechanical adaptability program. Biophysical J (2022) 121:3573–85. doi:10.1016/j.bpj.2022.04.039 PMC9617128 35505610

[11] Nelson CM. Mechanical control of cell differentiation: Insights from the early embryo. Annu Rev Biomed Eng (2022) 24:307–22. doi:10.1146/annurev-bioeng-060418-052527 35385680

[12] Bao G, Suresh S. Cell and molecular mechanics of biological materials. Nat Mater (2003) 2(11):715–25. doi:10.1038/nmat1001 14593396

[13] Campas O A toolbox to explore the mechanics of living embryonic tissues. Semin Cel Dev Biol (2016) 55:119–30. doi:10.1016/j.semcdb.2016.03.011 PMC4903887 27061360

[14] Kennedy BF, Wijesinghe P, Sampson DD. The emergence of optical elastography in biomedicine. Nat Photon (2017) 11(4):215–21. doi:10.1038/nphoton.2017.6

[15] Leartprapun N, Adie SG. Recent advances in optical elastography and emerging opportunities in the basic sciences and translational medicine [Invited]. Biomed Opt Express (2023) 14(1):208–48. doi:10.1364/boe.468932 36698669 PMC9842001

[16] Koski K, Yarger J. Brillouin imaging. Appl Phys Lett (2005) 87(6):061903. doi:10.1063/1.1999857

[17] Scarcelli G, Yun SH. Confocal Brillouin microscopy for three-dimensional mechanical imaging. Nat Photon (2008) 2(1):39–43. doi:10.1038/nphoton.2007.250 PMC2757783 19812712

[18] Zhang J, Scarcelli G. Mapping mechanical properties of biological materials via an add-on Brillouin module to confocal microscopes. Nat Protoc (2021) 16(2):1251–75. doi:10.1038/s41596-020-00457-2 33452504 PMC8218248

[19] Prevedel R, Diz-Muñoz A, Ruocco G, Antonacci G. Brillouin microscopy: An emerging tool for mechanobiology. Nat Methods (2019) 16(10):969–77. doi:10.1038/s41592-019-0543-3 31548707

[20] Palombo F, Fioretto D. Brillouin light scattering: Applications in biomedical sciences. Chem Rev (2019) 119(13):7833–47. doi:10.1021/acs.chemrev.9b00019 31042024 PMC6624783

[21] Poon C, Chou J, Cortie M, Kabakova I. Brillouin imaging for studies of micromechanics in biology and biomedicine: From current state-of-the-art to future clinical translation. J Phys Photon (2020) 3(1):012002. doi:10.1088/2515-7647/abbf8c

[22] Antonacci G, Beck T, Bilenca A, Czarske J, Elsayad K, Guck J, Recent progress and current opinions in Brillouin microscopy for life science applications. Biophysical Rev (2020) 12:615–24. doi:10.1007/s12551-020-00701-9 PMC7311586 32458371

[23] Boyd RW. Nonlinear optics. Academic Press (2003).

[24] Taylor MA, Kijas AW, Wang Z, Lauko J, Rowan AE. Heterodyne Brillouin microscopy for biomechanical imaging. Biomed Opt Express (2021) 12(10):6259–68 doi:10.1364/boe.435869 34745734 PMC8548004

[25] Scarponi F, Mattana S, Corezzi S, Caponi S, Comez L, Sassi P, High-performance versatile setup for simultaneous Brillouin-Raman microspectroscopy. Phys Rev X (2017) 7(3):031015. doi:10.1103/physrevx.7.031015

[26] Scarcelli G, Polacheck WJ, Nia HT, Patel K, Grodzinsky AJ, Kamm RD, Noncontact three-dimensional mapping of intracellular hydromechanical properties by Brillouin microscopy. Nat Methods (2015) 12(12):1132–4. doi:10.1038/nmeth.3616 26436482 PMC4666809

[27] Bevilacqua C, Sánchez-Iranzo H, Richter D, Diz-Muñoz A, Prevedel R. Imaging mechanical properties of sub-micron ECM in live zebrafish using Brillouin microscopy. Biomed Opt Express (2019) 10(3):1420–31. doi:10.1364/boe.10.001420 30891356 PMC6420298

[28] Rank D, Kiess EM, Fink U. Brillouin spectra of viscous liquids. JOSA (1966) 56(2): 163–6. doi:10.1364/josa.56.000163

[29] Xu J, Ren X, Gong W, Dai R, Liu D. Measurement of the bulk viscosity of liquid by Brillouin scattering. Appl Opt (2003) 42(33):6704–9. doi:10.1364/ao.42.006704 14658475

[30] Zhang J, Fiore A, Yun S-H, Kim H, Scarcelli G. Line-scanning Brillouin microscopy for rapid non-invasive mechanical imaging. Scientific Rep (2016) 6 : 35398. doi:10.1038/srep35398 PMC5064313 27739499

[31] Zhang J, Nikolic M, Tanner K, Scarcelli G. Rapid biomechanical imaging at low irradiation level via dual line-scanning Brillouin microscopy. Nat Methods (2023) 1–5. doi:10.1038/s41592-023-01816-Z 36894684 PMC10363327

[32] Bevilacqua C, Gomez JM, Fiuza U-M, Chan CJ, Wang L, Hambura S, High-resolution line-scan Brillouin microscopy for live-imaging of mechanical properties during embryo development. bioRxiv (2022) 2022. doi:10.1101/2022.04.25.489364 PMC10172129 36997817

[33] Rogers JA, Maznev AA, Banet MJ, Nelson KA. Optical generation and characterization of acoustic waves in thin films: Fundamentals and applications Annu Rev Mater Sci (2000) 30(1):117–57. doi:10.1146/annurev.matsci.30.1.117

[34] Nelson KA, Fayer M. Laser induced phonons: A probe of intermolecular interactions in molecular solids. J Chem Phys (1980) 72(9):5202–18. doi:10.1063/1.439756

[35] Gusev VE, Ruello P. Advances in applications of time-domain Brillouin scattering for nanoscale imaging. Appl Phys Rev (2018) 5(3):031101. doi:10.1063/1.5017241

[36] Kilicaslan I, Esen C, Schweiger G. Ultrasonic enhanced Brillouin light scattering in water. Opt Commun (2006) 265(2):441–5. doi:10.1016/j.optcom.2006.03.061

[37] Li T, Li F, Liu X, Yakovlev VV, Agarwal GS. Quantum-enhanced stimulated Brillouin scattering spectroscopy and imaging. Optica (2022) 9(8):959–64. doi:10.1364/optica.467635 PMC10312138 37398895

[38] Chiao R, Townes CH, Stoicheff B. Stimulated Brillouin scattering and coherent generation of intense hypersonic waves. Phys Rev Lett (1964) 12(21):592–5. doi:10.1103/physrevlett.12.592

[39] Remer I, Shaashoua R, Shemesh N, Ben-Zvi A, Bilenca A. High-sensitivity and high-specificity biomechanical imaging by stimulated Brillouin scattering microscopy. Nat Methods (2020) 17(9):913–6. doi:10.1038/s41592-020-0882-0 32747769

[40] Zanini G, Scarcelli G. Localization-assisted stimulated Brillouin scattering spectroscopy. APL Photon (2022) 7(5):056101. doi:10.1063/5.0087697 PMC9073852 35547354

[41] Remer I, Cohen L, Bilenca A. High-speed continuous-wave stimulated Brillouin scattering spectrometer for material analysis. JoVE (Journal of Visualized Experiments) (2017)(127) e55527. doi:10.3791/55527-v PMC5752319 28994794

[42] Nikolić M, Scarcelli G. Long-term Brillouin imaging of live cells with reduced absorption-mediated damage at 660nm wavelength. Biomed Opt Express (2019) 10(4): 1567–80. doi:10.1364/boe.10.001567 31086695 PMC6484981

[43] Ballmann CW, Thompson JV, Traverso AJ, Meng Z, Scully MO, Yakovlev VV. Stimulated brillouin scattering microscopic imaging. Scientific Rep (2015) 5:18139. doi:10.1038/srep18139 PMC4686920 26691398

[44] Yang F, Bevilacqua C, Hambura S, Neves A, Gopalan A, Watanabe K, Pulsed stimulated Brillouin microscopy enables high-sensitivity mechanical imaging of live and fragile biological specimens. bioRxiv (2022). 10.1038/s41592-023-02054-z PMC10703689 37884795

[45] Remer I, Bilenca A. Background-free Brillouin spectroscopy in scattering media at 780 nm via stimulated Brillouin scattering. Opt Lett (2016) 41(5):926–9. doi:10.1364/ol41.000926 26974082

[46] Barer R, Ross K, Tkaczyk S. Refractometry of living cells. Nature (1953) 171(4356) 720–4. doi:10.1038/171720a0 13054689

[47] Schürmann M, Scholze J, Müller P, Guck J, Chan CJ. Cell nuclei have lower refractive index and mass density than cytoplasm. J biophotonics (2016) 9(10):1068–76 doi:10.1002/jbio.201500273 27010098

[48] Schlüßler R, Möllmert S, Abuhattum S, Cojoc G, Müller P, Kim K, Mechanical mapping of spinal cord growth and repair in living zebrafish larvae by brillouin imaging. Biophysical J (2018) 115(5):911–23. doi:10.1016/j.bpj.2018.07.027 PMC6127462 30122291

[49] Nelson KA, Miller RD, Lutz D, Fayer M. Optical generation of tunable ultrasonic waves. J Appl Phys (1982) 53(2):1144–9. doi:10.1063/1.329864

[50] Yan YX, Nelson KA. Impulsive stimulated light scattering. I. General theory. J Chem Phys (1987) 87(11):6240–56. doi:10.1063/1.453733

[51] Yan YX, Nelson KA. Impulsive stimulated light scattering. II. Comparison to frequency-domain light-scattering spectroscopy. J Chem Phys (1987) 87(11):6257–65. doi:10.1063/1.453454

[52] Shen Y-c., Hess P. Real-time detection of laser-induced transient gratings and surface acoustic wave pulses with a Michelson interferometer. J Appl Phys (1997) 82(10): 4758–62. doi: 10.1063/1.366332

[53] Miller RD, Casalegno R, Nelson KA, Fayer M. Laser-induced ultrasonics: A daynamic holographic approach to the measurement of weak absorptions, optoelastic constants acoustic attenuation. Chem Phys (1982) 72(3):371–9. doi:10.1016/03010104(82)85134-3

[54] Robinson MM, Yan Y-X, Gamble EB Jr, Williams LR, Meth JS, Nelson KA Picosecond impulsive stimulated brillouin scattering: Optical excitation of coherent transverse acoustic waves and application to time-domain investigations of structural phase transitions. Chem Phys Lett (1984) 112(6):491–6. doi:10.1016/0009-2614(84)85764-4

[55] Krug B, Koukourakis N, Guck J, Czarske J. Nonlinear microscopy using impulsive stimulated Brillouin scattering for high-speed elastography. Opt express (2022) 30(4): 4748–58. doi:10.1364/oe.449980 35209449

[56] Kinoshita S, Shimada Y, Tsurumaki W, Yamaguchi M, Yagi T. New high-resolution phonon spectroscopy using impulsive stimulated Brillouin scattering. Rev scientific Instr (1993) 64(12):3384–93. doi:10.1063/1.1144309

[57] Rogers JA, Fuchs M, Banet MJ, Hanselman JB, Logan R, Nelson KA. Optical system for rapid materials characterization with the transient grating technique: Application to nondestructive evaluation of thin films used in microelectronics. Appl Phys Lett (1997) 71(2):225–7. doi:10.1063/1.119506

[58] Maznev A, Nelson K, Rogers J. Optical heterodyne detection of laser-induced *gratings*. Opt Lett (1998) 23(16):1319–21. doi:10.1364/ol.23.001319 18087511

[59] Li J, Zhang H, Chen X, Le T, Wei H, Li Y. High-speed non-contact measurement of elasto-optic coefficient via laser-induced phonons. Appl Phys Lett (2022) 121(25): 251102. doi:10.1063/5.0134976

[60] Meng Z, Petrov GI, Yakovlev VV. Flow cytometry using Brillouin imaging and sensing via time-resolved optical (BISTRO) measurements. Analyst (2015) 140(21): 7160–4. doi:10.1039/c5an01700a 26347908 PMC5642965

[61] Ballmann CW, Meng Z, Traverso AJ, Scully MO, Yakovlev VV. Impulsive brillouin microscopy. Optica (2017) 4(1):124–8. doi:10.1364/optica.4.000124

[62] Krug B, Koukourakis N, Czarske JW. Impulsive stimulated Brillouin microscopy for non-contact, fast mechanical investigations of hydrogels. Opt express (2019) 27(19): 26910–23. doi:10.1364/oe.27.026910 31674562

[63] Li J, Zhang H, Lu M, Wei H, Li Y. Sensitive impulsive stimulated Brillouin spectroscopy by an adaptive noise-suppression Matrix Pencil. Opt express (2022) 30(16):29598–610. doi:10.1364/oe.465106 36299131

[64] Caponi S, Fioretto D, Mattarelli M. On the actual spatial resolution of Brillouin Imaging. Opt Lett (2020) 45(5):1063–6. doi:10.1364/ol.385072 32108770

[65] Thomsen C, Strait J, Vardeny Z, Maris HJ, Tauc J, Hauser J. Coherent phonon generation and detection by picosecond light pulses. Phys Rev Lett (1984) 53(10): 989–92. doi:10.1103/physrevlett.53.989

[66] Thomsen C, Grahn HT, Maris HJ, Tauc J. Surface generation and detection of phonons by picosecond light pulses. Phys Rev B (1986) 34(6):4129–38. doi:10.1103/physrevb.34.4129 9940178

[67] Pérez-Cota F, Smith RJ, Moradi E, Marques L, Webb KF, Clark M. High resolution 3D imaging of living cells with sub-optical wavelength phonons. Scientific Rep (2016) 6(1):39326–11. doi:10.1038/srep39326 PMC5171858 27996028

[68] Danworaphong S, Tomoda M, Matsumoto Y, Matsuda O, Ohashi T, Watanabe H, Three-dimensional imaging of biological cells with picosecond ultrasonics. Appl Phys Lett (2015) 106(16):163701. doi:10.1063/1.4918275

[69] Smith RJ, Pérez-Cota F, Marques L, Clark M. 3D phonon microscopy with sub-micron axial-resolution. Scientific Rep (2021) 11(1):3301. doi:10.1038/s41598-02182639-w PMC7870650 33558575

[70] Rossignol C, Chigarev N, Ducousso M, Audoin B, Forget G, Guillemot F, In vitro picosecond ultrasonics in a single cell. Appl Phys Lett (2008) 93(12):123901. doi:10.1063/1.2988470

[71] Audoin B, Rossignol C, Chigarev N, Ducousso M, Forget G, Guillemot F, Picosecond acoustics in vegetal cells: Non invasive in vitro measurements at a sub-cell scale. Phys Proced (2010) 3(1):323–31. doi:10.1016/j.phpro.2010.01.043 19879618

[72] Dehoux T, Abi Ghanem M, Zouani OF, Ducousso M, Chigarev N, Rossignol C, Probing single-cell mechanics with picosecond *ultrasonics*. Ultrasonics (2015) 56 : 160–71. doi:10.1016/j.ultras.2014.07.010 25172112

[73] Dehoux T, Ghanem MA, Zouani O, Rampnoux J-M, Guillet Y, Dilhaire S, All-optical broadband ultrasonography of single cells. Scientific Rep (2015) 5(1):8650–5 doi:10.1038/srep08650 PMC4346798 25731090

[74] Pérez-Cota F, Smith RJ, Moradi E, Marques L, Webb KF, Clark M. Thin-film optoacoustic transducers for subcellular Brillouin oscillation imaging of individual biological cells. Appl Opt (2015) 54(28):8388–98. doi:10.1364/ao.54.008388 26479614

[75] Pérez-Cota F, Smith RJ, Elsheikha HM, Clark M. New insights into the mechanical properties of Acanthamoeba castellanii cysts as revealed by phonon microscopy. Biomed Opt express (2019) 10(5):2399–408. doi:10.1364/boe.10.002399 31143495 PMC6524581

[76] La Cavera S III, Pérez-Cota F, Smith RJ, Clark M. Phonon imaging in 3D with a fibre probe. Light: Sci Appl (2021) 10(1):91. doi:10.1038/s41377-021-00532-7 33907178 PMC8079419

